# The Neuroanatomic Diagnostic Protocol: Evidence for a Necessary Update to the Clinical Framework for Managing Chronic Cough

**DOI:** 10.1007/s00408-026-00959-y

**Published:** 2026-11-13

**Authors:** Stuart Mazzone, Surinder S. Birring, Peter G. Gibson, Woo-Jung Song

**Affiliations:** 1 Department of Anatomy and Physiology, University of Melbourne, Melbourne, VIC Australia; 2 Centre for IMPACT Chronic Cough, NHMRC Centre of Research Excellence, University of Melbourne, Melbourne, VIC Australia; 3 Faculty of Life Sciences and Medicine, Centre for Human and Applied Physiological Sciences, School of Basic and Medical Biosciences, King’s College London, London, UK; 4 Asthma and Breathing Research Program, Hunter Medical Research Institute, The University of Newcastle, Newcastle, 2308 NSW Australia; 5 Respiratory & Sleep Medicine Department, John Hunter Hospital, Newcastle, 2305 NSW Australia; 6 University of Ulsan College of Medicine, Seoul, Korea; 7 Department of Allergy and Clinical Immunology, Asan Medical Center, Seoul, Korea

**Keywords:** Vagus nerve, Hypersensitivity, Paresthesia, Antitussive, Refractory chronic cough, Cough triggers

## Abstract

**Background:**

Chronic cough represents a challenging clinical problem and significant unmet global burden. For some patients, cough can be associated with conditions impacting the respiratory system, including asthma, rhinosinusitis, and gastroesophageal reflux disease. This underpinned the rationale for the development of the Anatomic Diagnostic Protocol, an etiology-based algorithm for chronic cough management used in clinical practice for over 50 years. However, despite the value of the Anatomic Diagnostic Protocol, many patients do not respond adequately to the clinical strategies promoted in the existing management framework because they either have no obvious causative disease or their cough is unresolved by standard etiology-based therapies (refractory chronic cough). This, along with the introduction of cough hypersensitivity syndrome as a unifying condition common to most patients with chronic cough, has added additional complexity for physicians in everyday practice.

**Findings:**

We introduce the Neuroanatomic Diagnostic Protocol and the rationale behind it, a proposed timely and practical update to the classic framework that unifies the known association of cough with common etiological causes, the existence of refractory chronic cough, and the core concepts of cough hypersensitivity syndrome by acknowledging the vagus nerve and its central circuits as a distinct anatomical consideration in the effective assessment and targeted management of chronic cough.

## Introduction

Chronic cough is defined as cough persisting for longer than 8 weeks in adults and represents a challenging clinical problem for both physicians and patients. Epidemiological studies estimate the global prevalence of chronic cough to be 6–10% of the adult population, with many individuals enduring cough for years or decades of their life despite extensive healthcare interventions [1, 2]. For some patients, cough can be associated with a range of conditions impacting the respiratory system, including asthma, chronic obstructive pulmonary disease (COPD), interstitial lung disease, bronchiectasis, nasal disease and gastroesophageal reflux disease (GERD). However, in a significant number of individuals no obvious associations with a potential causative disease or disorder can be identified despite extensive clinical investigation [3]. Furthermore, for many patients with an identifiable disorder known to precipitate cough, their chronic cough remains persistent despite effective therapeutic management of the presumptive causative condition [4]. Identifying management strategies for patients who do not respond to common etiology-targeted therapies (defined as refractory chronic cough) remains a significant clinical challenge, especially considering a long-standing absence of safe and effective primary cough suppression drugs for physicians to prescribe [4].

The last decade has witnessed a rapid acceleration in our understanding of chronic cough. Mechanistic studies have provided insight into the neurobiological dysfunctions and pathologies that exist in individuals with chronic cough [5], and drug development programs are finally progressing new therapies that target these neural mechanisms [6]. However, this does not mean that past learnings and practices should now be discounted or discontinued. Instead, there is an opportunity to review and revise the existing diagnostic and treatment foundations for chronic cough, to incorporate the new understandings and treatment opportunities in development. Here we review the literature to develop a case for a timely and practical update to the Anatomic Diagnostic Protocol [7], a longstanding and widely utilized framework for chronic cough management. We first explore the strengths and limitations of the Anatomic Diagnostic Protocol and there role of common diseases in precipitating altered vagal function. We also review the concept of cough hypersensitivity from the perspective of a primary disorder of vagal function. Lastly, we bring these concepts together to synthesise a new approach for managing patients with chronic cough. Our premise is that a rationale exists to retain the core elements of the Anatomic Diagnostic Protocol while additionally recognizing the nervous system as an important and targetable organ that should be assessed and considered as part of a revised standard clinical algorithm, the *Neuroanatomic Diagnostic Protocol*, for managing patients with chronic cough.

## The Original Anatomic Diagnostic Protocol

### Historical Overview

**Fig. 1:**
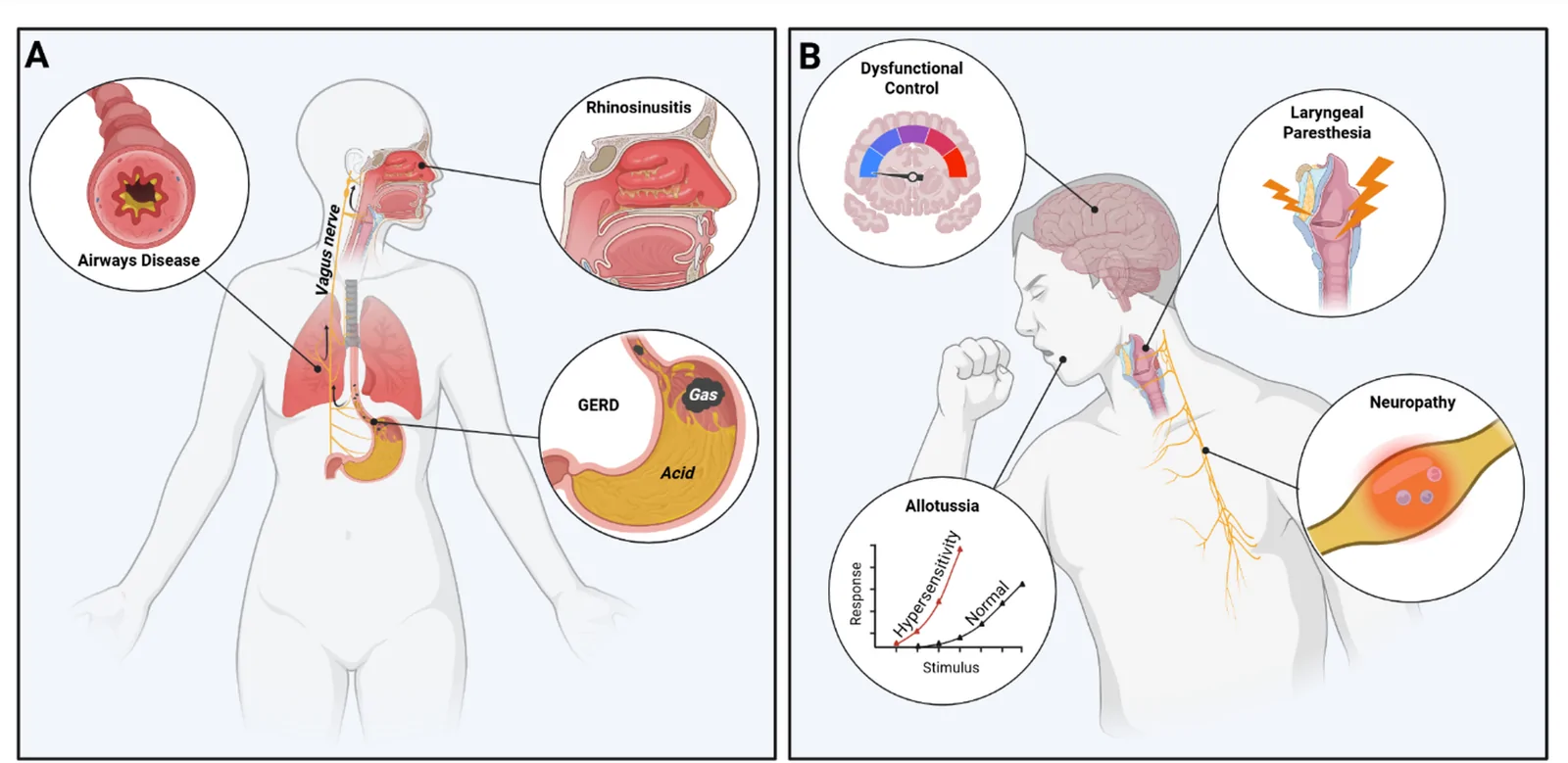
An overview of the two fundamental frameworks that assist in the understanding and management of chronic cough in adults. **A** Etiology-based approaches propose that because of the anatomical distribution of the vagus nerve, patients who develop chronic cough caused by diseases of the nose, esophagus and airways, do so due to an impact of the disease on vagal functioning. In such cases, the resultant vagal dysfunction is secondary to the underlying disease. The evidence supporting an etiology-based assessment and management approach is that patients with chronic cough may respond favorably to therapies targeting diseases of these organs. **B** Cough hypersensitivity syndrome proposes that chronic cough is accompanied by heightened responsiveness to chemical, mechanical and thermal stimuli due to disordered functioning or disease of the vagus nerve and its central circuits, processes that may be especially responsible for chronic cough in patients with no explainable disease or patients who have an identifiable associated disease but their cough is either partially or fully refractory to common etiology-based therapies. In this case, cough may be a primary disorder of vagal functioning, independent of any comorbid pathologies that also exist. The evidence supporting this includes the presentation of hypertussia, allotussia, and laryngeal paresthesia in many patients with chronic cough and their clinical responsiveness to neuromodulators or cough behavioural therapies. The Neuro-Anatomical Diagnostic Protocol unifies these frameworks, proposing that airways disease, nasal disease, esophageal disease and primary vagal neural hypersensitivity be considered as four treatable traits in the wholistic management of chronic cough. Created using Biorender

The Anatomic Diagnostic Protocol for chronic cough was developed in the late 1970s, primarily through the work of Irwin and colleagues [7, 8]. The protocol represented a major conceptual shift in the approach to cough management. Instead of treating cough as a nonspecific symptom, it proposed that chronic cough could usually be traced to identifiable diseases affecting the anatomic distribution of cough receptors and their afferent (sensory) neural pathways [8] (Fig. 1). The central premise was that careful, systematic evaluation of patients followed by etiology-targeted therapy could resolve cough in most patients. The first major description appeared in 1981, when Irwin et al. [7] prospectively evaluated patients with chronic persistent cough using a structured diagnostic approach based on anatomy and known vagal cough receptor distribution. In 49 consecutive and unselected patients, a specific diagnosis was reportedly made in all patients, with treatment success rates approaching 98%. This and subsequent work provided evidence that, in nonsmokers with normal chest radiographs and no ACE inhibitor use, the overwhelming majority of chronic cough could be explained by upper airway (nasal) disease (synonymous with postnasal drip syndrome or upper airway cough syndrome), asthma or eosinophilic airway disease, and GERD [7, 9, 10]. The concept of a “pathogenic triad” of asthma, postnasal drip syndrome, and GERD then became well established (Fig. 1), noting that these conditions alone or in combination accounted for more than 90% of chronic cough cases in specialised clinics [11, 12]. Historically, this work was highly important because chronic cough had previously been regarded as diagnostically frustrating symptom and often ‘idiopathic’. Accordingly, its evolution continued into a sequential algorithm and informed clinical guidelines [13, 14] that included a detailed history and physical examination, chest radiography, assessment for upper airway disease, bronchoprovocation testing (e.g., methacholine challenge) for occult asthma, empiric therapeutic trials for the common causative conditions, sinus imaging or rhinoscopy when indicated, and 24-hour oesophageal pH monitoring for suspected reflux-associated cough.

### Emergent Limitations in the Anatomic Diagnostic Protocol

Despite the major influence of the Anatomic Diagnostic Protocol on chronic cough management, limitations gradually emerged as the approach underwent broader scrutiny. One concern was that the remarkably high success rates initially reported were derived predominantly from uncontrolled observational studies conducted in highly specialised tertiary cough clinics, while independent investigators often failed to reproduce the reported success outcomes in more heterogeneous clinical populations. Poe and colleagues [15] reported that although potential causes could be identified in approximately 88% of patients, therapeutic responses were less predictable and successful than originally proposed. Critics also highlighted the absence of randomized controlled trials validating the sequential empiric treatment strategy, referral-centre bias toward selected patient populations, and a circular diagnostic reasoning in which improvement after therapy was itself used to retrospectively confirm the diagnosis. This approach made it difficult to distinguish true causal mechanisms from placebo effects, spontaneous resolution, regression to the mean, or nonspecific therapeutic responses, which are all now recognized as common confounds with significant influence in cough clinical trials [16].

GERD became one of the most controversial aspects of the Anatomic Diagnostic Protocol. Early protocol-based studies suggested that empiric proton pump inhibitor (PPI) therapy produced substantial clinical improvement, even in the absence of classical GERD symptoms [17]. However, subsequent PPI placebo-controlled trials demonstrated more modest benefit, which was only observed in patients with peptic symptoms [18–20]. These findings challenged the assumption that acid reflux was a major causal driver of chronic cough and weakened the recommendation for an empirical trial of PPIs in international cough guidelines [21, 22]. The diagnosis of postnasal drip syndrome has similarly been challenged, due to a lack of objective diagnostic criteria, instead relying on subjective symptoms such as throat clearing, nasal discharge, or pharyngeal irritation, or responsiveness to 1st generation sedating antihistamines which are also known to be antitussive [13, 23–25]. This uncertainty in the existence of postnasal drip syndrome prompted a shift in terminology toward “upper airway cough syndrome” reflecting growing recognition that the mechanisms linking upper airway disease to chronic cough remained poorly defined and potentially non-causal. Nevertheless, whether upper airway diseases such as allergic rhinitis or rhinosinusitis represent true causal conditions or treatable traits in chronic cough still remains unresolved [26].

Despite the uncertainties, the Anatomic Diagnostic Protocol remains widely utilized as a clinical framework because of its pragmatic utility. However, reports indicate that up to 40% of patients may not respond adequately to the clinical strategies promoted in the existing management framework [27, 28]. As exemplified by the examples of GERD and upper airway cough syndrome, these uncertainties often perpetuate a cycle of repeated empirical therapeutic trials, in the absence of etiology-specific symptoms or objective diagnostic evidence. As cough may wax and wane spontaneously or independently of treatment, symptomatic improvement may be incorrectly interpreted as diagnostic confirmation, resulting in prolonged healthcare journeys without a clear explanation of the patient’s cough. Such diagnostic uncertainty may itself become a source of disease burden, prolonging healthcare journeys and delaying effective management while contributing to anxiety, depression, frustration, and impaired quality of life among patients [29, 30], while increasing healthcare utilization, unnecessary medications with risks of adverse effects, and socioeconomic costs [31–35].

## Evidence for Primary Vagal Neural Dysfunction as a Cause of Chronic Cough

### Cough Hypersensitivity

The concept that most patients with chronic cough may be considered as having a single diagnosis, cough hypersensitivity syndrome, fundamentally challenged the understanding of chronic cough [36–38]. Rather than viewing chronic cough as an exaggerated protective reflex that manifests in some patients secondary to diseases such as rhinosinusitis, asthma or GERD, the possible expression of cough hypersensitivity as a common underlying trait in patients who develop cough reframed the disorder as a manifestation of sensory neural dysfunction [4]. Cough hypersensitivity is broadly defined as troublesome coughing triggered by low levels of thermal, mechanical, or chemical exposure, reflecting an abnormal change in the responsiveness of the neural pathways responsible for regulating cough [38]. This paradigm shift emerged alongside the recognition that chronic cough in many patients can be refractory to therapies targeting specific associated diseases, while also demonstrating heightened sensitivity to tussive agents and innocuous environmental stimuli. The concept has been further progressed to acknowledge that the clinical features of hypersensitivity include the expression of abnormal sensations, along with evidence of both hypertussia and allotussia [39, 40] (Fig. 1). Hypertussia describes an exaggerated cough response to recognised tussive stimuli such as cigarette smoke, fumes, aerosols, or bleach, while allotussia refers to coughing provoked by stimuli that would not normally induce cough, including talking, laughing, eating, exposure to perfumes, changes in air temperature, or even light pressure on the neck or chest. These overexaggerated expressions of cough were so named as they remarkably resembled the hyperalgesia and allodynia commonly seen in patients with chronic pain hypersensitivity syndromes [40–42].

More than 95% of patients with chronic cough report an abnormal sensitivity to environmental triggers accompanied by cough and a persistent urge-to-cough sensation [43]. Accordingly, cough hypersensitivity is now widely regarded as a unifying clinical feature of chronic cough irrespective of the initiating etiology. Many patients specifically exhibit features of laryngeal hypersensitivity, including paradoxical vocal fold movement, muscle tension dysphonia, throat irritation, and globus sensation, suggesting that the larynx functions as a key site of aberrant sensory processing [43, 44]. Indeed, the larynx receives perhaps the densest vagal sensory innervation and represents a high reflexogenic site within the airways. Hypersensitivity may additionally extend more broadly across the territories of the vagus nerve, as approximately 25% of patients with cough hypersensitivity also displays a positive Arnold’s nerve cough reflex, triggered by mechanical stimulation to the external auditory canal where the auricular branch of the vagus nerve terminates [45]. This is contrast to approximately 2% of healthy individuals without chronic cough having a positive Arnold’s nerve reflex [45].

Preclinical and clinical studies now strongly support this concept, demonstrating that cough hypersensitivity involves both peripheral and central neural mechanisms, including the sensitization of vagal afferent pathways, altered brainstem processing, and dysfunction within higher cortical networks governing cough perception and suppression [5, 39] (Fig. 1). Inflammatory insults, viral infections, environmental irritants, and potentially genetic susceptibility are all believed to contribute to aberrant neural responsivity, injury or maladaptive neuroplasticity within cough pathways. For example, in animal models C-fibres appear to play a particularly important role in both abnormal vagal sensory processing and chronic cough [46]. These fibres mediate nociceptive and irritant signalling, through their expression of a suite of receptors and ion channels which transduce noxious signals into action potentials for transmission to the central neural circuits involved in cough generation. Additionally, C-fibres are highly susceptible to becoming sensitized and undergo structural remodelling following inflammation or neural injury, and this is believed to contribute to excessive activation and the generation of aberrant sensations and a persistent urge-to-cough [47]. Changes in central neural circuits may also be involved in chronic cough. In humans, morphometric studies have identified changes to regional grey matter thickness in the brains of patients with refractory chronic cough [48]. Furthermore, cough hypersensitivity is associated with exaggerated neural activity in the midbrain coupled with a loss of activity in central neural systems needed for cough suppression [49, 50]. Consequently, even low-level sensory inputs to the central nervous system may be sufficient to evoke coughing due to alterations in the coupling of sensory inputs into motor actions [50] (Fig. 1). Accordingly, we now recognize cough hypersensitivity as a form of neural hypersensitivity, which provides a new mechanistic opportunity to target therapeutically in chronic cough.

### Laryngeal and Chest Paresthesia

The morbidity associated with chronic cough is not only caused by the act of coughing but also by the inability to satiate persistent abnormal throat or chest sensations [43, 51]. Paresthesia refers to abnormal sensory experiences that occur either spontaneously or in response to stimuli that would not ordinarily provoke significant sensation. In neurology, paresthesia classically includes tingling, burning, itching, crawling, electric-shock-like feelings, or the well-known sensation of “pins and needles” [52]. Disorders such as diabetic neuropathy, postherpetic neuralgia, multiple sclerosis, trigeminal neuralgia, and small fibre neuropathy all produce abnormal sensory experiences through mechanisms involving peripheral nerve injury, demyelination, sensory ganglion dysfunction, abnormal ion channel activity, or central sensitisation. Thus, the biological importance of paresthesia lies in the fact that it reflects aberrant nervous system activity. Notably, when sensory pathways become damaged, inflamed, sensitized, or structurally altered, neurons may begin to fire spontaneously or excessively, generating sensations in the absence of appropriate peripheral stimuli. In many neurological disorders, paresthesia therefore serves as a clinical marker of neural hyperexcitability [53, 54].

The presence of laryngeal or chest paresthesia is evidence of aberrant sensory neural processing involving vagal afferent pathways [4, 5, 55, 56]. Patients with chronic cough commonly describe a wide range of abnormal throat or chest sensations including tickling, irritation, burning, itching, dryness, a sense of obstruction and chest tightness or pressure [43, 51, 57]. These sensations are remarkably stereotyped across geographically and clinically distinct patient populations, suggesting a shared neurobiological mechanism. They are frequently localised to the throat (larynx or adjacent upper airway) but additionally can be experienced in the chest and often immediately precede coughing episodes due an escalating experience of an urge-to-cough. Indeed, laryngeal paresthesia may be the primary symptom of this condition, whereas cough is a secondary phenomenon to relieve the sensation. In support of this, many patients describe an intrusive sensory experience that progressively intensifies until coughing occurs, after which temporary relief may be experienced until the cycle of increasing irritation and urge begins again [58]. Importantly, these sensory experiences often occur in the absence of major laryngeal or airway structural pathology and resemble the expression of paresthesia associated with neuropathic mechanisms observed elsewhere in the nervous system. For example, in neuropathic itch syndromes, patients experience persistent itching or crawling dermal sensations despite absent dermatological pathology, generating an intense urge-to-scratch and resultant scratching behaviours that provide only temporary relief before the itch-scratch cycle begins again [59]. This is a striking parallel with laryngeal paraesthesia, urge-to-cough and cough behaviours in patients with chronic cough. In this regard, cough and frequent throat clearings may reflect a behavioral attempt to clear or ‘scratch’ the throat or relieve the airway sensations, which are often described as itching or a tickle by patients, caused by dysfunctional vagal sensory processing.

### Response to Neuromodulators

Neuromodulator therapies are an effective option for managing chronic cough in some patients [21, 60, 61]. Opiate drugs have long been known to be effective cough suppressants [21, 62, 63], while other centrally acting agents such as gabapentin, pregabalin, amitriptyline, and nortriptyline have also demonstrated benefit, especially in refractory chronic cough [64–66]. For example, clinical trials of gabapentin and low-dose morphine in refractory chronic cough have demonstrated improvements in cough-specific quality of life, reductions in cough frequency, and decreases in cough severity [62, 67, 68]. Importantly, these benefits are often accompanied by reductions in laryngeal irritation and urge-to-cough sensations, suggesting that the therapies are not simply acting by suppressing motor cough output but also by modulating the abnormal sensory experiences that precede coughing [62]. This distinction is critical because it implies that the primary therapeutic target is altered sensory processing rather than airway mechanics or cough motor controls. Indeed, these drugs are not known to have any direct anti-inflammatory or bronchodilatory effects and neuromodulators tend to be most effective in patients with prominent paresthesia.

The mechanisms of action of central neuromodulators are best characterized in neuropathic pain syndromes, including diabetic neuropathy, postherpetic neuralgia, trigeminal neuralgia, and fibromyalgia, where they have been shown to reduce neural excitability and dampen abnormal sensory signalling in the brain [54]. Their mechanisms of action converge on the reduction of sensory gain, acting to reduce signalling involved in encoding pain sensations. Gabapentin and pregabalin, for example, reduce cough by bind the α2δ subunit of voltage-gated calcium channels, reducing excitatory neurotransmitter release within central nervous system sensory pathways [54]. Tricyclic antidepressants such as amitriptyline and nortriptyline reduce cough by mimicking the descending inhibitory (analgesic) pathways through effects on serotonin and noradrenaline signalling which suppress sensory nerve inputs to the central nervous system [69]. Opiate drugs reduce cough likely act a multiple sites along central nociceptive pathways, including mimicking the action of endogenous opioids in enhancing the descending analgesia system [70]. Additionally, the responsiveness of chronic cough to behavioral neuromodulation, such as speech pathology-based cough suppression therapy [71], suggests that cough hypersensitivity involves maladaptive neural plasticity across both peripheral and central pathways [4, 72]. Behavioral therapies designed to suppress cough may act by re-engaging central voluntary suppression mechanisms that are diminished in chronic cough [49], thereby serving as a form of sensory-motor retraining that alters attentional processing, laryngeal control, and cortical modulation of sensory inputs.

### Vagal Neuropathies

The notion of vagal neuropathy may underlie chronic cough in some patients is not new, albeit this diagnosis has mostly been speculative based on clinical presentation, typically in post-viral cough where neuropathy is presumed to be the cause of laryngeal dysfunction [39, 40, 73, 74]. For example, the persistent throat tickling, laryngeal irritation, and heightened sensitivity to environmental triggers long after resolution of some respiratory viral infections is similar to the symptom profile in well-established viral-induced sensory neuropathies that impact somatosensory systems (e.g., post-herpetic and trigeminal neuralgias) [40]. Preclinical studies in animals provides some support for this pathogenic framework, albeit relatively severe viral infections are needed to generate significant neuropathic effects in the vagal nerves [75]. Nevertheless, the empirical evidence for neuropathic processes and vagal injury in any type of chronic cough in humans has been, until recently, limited.

Perhaps the most compelling evidence supporting a neuropathic basis for chronic cough is in the case of the genetic condition Cerebellar Ataxia, Neuropathy, and Vestibular Areflexia Syndrome (CANVAS) [76, 77], a late-onset neurodegenerative disorder caused by a biallelic intronic pentanucleotide repeat expansions in the replication factor C subunit 1 (RFC1) gene. Chronic cough is frequently identified in patients with CANVAS, with incidence rates perhaps as high as 97% [76]. Interestingly, chronic cough in these patients commonly precedes other neurological manifestations by decades, suggesting that vagal sensory dysfunction may be among the earliest presentations of RFC1-related disease. Consequently, patients likely seek healthcare solutions for their cough, long before other neurological signs manifest, making it challenging for clinicians to recognise CANVAS cough from other forms of refractory chronic cough. In this regard, the recent recognition that monoallelic and biallelic RFC1 repeat expansions are enriched among patients with refractory or unexplained chronic cough, even in the absence of overt ataxia [78–80], has broadened the phenotypic spectrum of RFC1 disease and raises many questions about the potential prevalence of genetic-based neuropathies in patients with difficult to treat chronic cough.

The pathological features of CANVAS neuropathy are best described as a sensory ganglionopathy in which large diameter sensory neurons progressively die causing the axonal content of peripheral nerves to degenerate [76]. Peripheral nerve conduction studies reflect this with a loss of sensory nerve action potential conduction, while imaging and histological studies confirm vagal hypotrophy and peripheral tissue sensory neuron denervation (including in the airways) [76, 81, 82]. The exact pathogenic mechanism of the RFC1 repeat expansion remains unclear, although RFC1 protein is involved in DNA replication and repair and current evidence suggests a toxic gain-of-function or RNA-mediated neurodegenerative process selectively affecting large sensory neurons, cranial sensory ganglia, vestibular pathways, and autonomic fibers. Yet despite this loss of sensory nerve fibers, chronic cough in patients with CANVAS similarly presents as cough hypersensitivity [81]. Exactly why peripheral sensory denervation leads to cough hypersensitivity is unknown, but prevailing hypotheses suggest a role for altered excitability of preserved sensory fibers or induction of centrally mediated denervation hypersensitivity, similar to that in some denervation-induced pain syndromes [76].

Another intriguing, albeit controversial, recent addition to possible neuropathic causes of chronic cough is VANCOUVER syndrome (Vagus Associated Neurogenic Cough Occurring due to Unilateral Vascular Encroachment of the Root) [83–85]. This condition was proposed following the observation of a patient with refractory chronic cough with vascular compression of the vagus nerve root entry zone at the level of the brainstem, analogous to the vascular compression syndromes observed in trigeminal neuralgia and hemifacial spasm. Vascular compression of vagal afferent fibres at the brainstem is proposed to produce aberrant sensory neuron firing, leading to neuropathic cough hypersensitivity. Impressively, complete cough resolution was reported for this patient following microvascular decompression surgery [83]. Although the prevalence of VANCOUVER syndrome in chronic cough remains to be clarified, and it may ultimately represent a rare cough endotype, its existence highlights the potential of direct vagal injury and neuropathic processes to precipitate chronic cough and the need to consider primary vagal neural hypersensitivity in cough diagnostic and management protocols.

## The revised Neuroanatomic Diagnostic Protocol

The original Anatomic Diagnostic Protocol already acknowledges the importance of appreciating the underlying neuroanatomy in chronic cough, ascribing cough as vagal condition that can manifest secondary to an associated pathological process in the airways, nose or gut [7, 9]. Our proposed update expands this definition and incorporates the concept of cough hypersensitivity into a unified construct that acknowledges the vagus nerve itself and its recipient central pathways as an important and targetable primary anatomic consideration in the diagnosis of chronic cough, especially in managing chronic cough that is refractory to standard interventions. The new protocol incorporates recommended clinical features and tools that enable assessment of primary vagal hypersensitivity concurrently alongside that of the standard assessment of other common etiology-based causes of chronic cough, potentially facilitating more timely identification of refractory cough. Furthermore, we propose that by recognizing the indispensable roles of the peripheral and central nervous systems in chronic cough through the revision of the protocol’s name will help refocus clinical action towards appropriately assessing and managing the both the primary and secondary neural underpinnings of the disorder.

### Assessing and Managing Primary Vagal Neural Hypersensitivity

Clinicians are generally accustomed to assessing patients for asthma/ eosinophilic airways disease, GERD or nasal disease, which remain common, albeit not ubiquitous, causes of chronic cough. By contrast, the identification of refractory chronic cough has proven more challenging as currently this label is often applied solely as a diagnosis of exclusion. Thus, only after the failure of extensive trials, and often retrials, of etiology-targeted therapies is a patient diagnosed as having a cough that is refractory [3, 21]. In fact, many clinicians may never reach this diagnosis as it can be seen as a failure to find the ‘true’ underlying cause of chronic cough, which is equally unsatisfying for patients and clinicians alike. Yet, with the evidence presented above, the underlying cause is highly likely to be primary vagal neural hypersensitivity, the recognition of which establishes a positive pathway forward for targeted management. Indeed, there are current therapeutic options at hand, and emerging therapies progressing along development pipelines, to consider for patients with this differential diagnosis [3, 4, 21].

The acceptance of primary vagal neural hypersensitivity as a cause of chronic cough raises the question of how to diagnose this condition. Unfortunately, no definitive biomarkers or clinical tests exist yet, and therefore the diagnosis relies on a combination of robust clinical investigation, which may include therapeutic trials for the common identifiable causative comorbidities advocated in the existing Anatomic Diagnostic Protocol. Chest imaging (preferably CT-imaging if history, physical exam and lung function testing do not help elucidate the diagnosis) remains a foundational cornerstone of any cough diagnostic work-up to appropriately identify and manage radiological red flags [3, 21]. Modern guidelines exist to help minimize repetitive trials of treatment and reliance on them, while also encouraging consideration of cough hypersensitivity [3]. Patients with obvious signs of asthma or eosinophilic airways disease (reversible airway obstruction, airways hyperresponsiveness, elevated FeNO etc.), GERD or esophageal dysmotility (heart burn, regurgitation, positive esophageal pH impedance etc.), or nasal disease (rhinitis, sinusitis etc.) should progress on to a trial of targeted therapy for those conditions [3, 13, 21]. But equally, a careful assessment of patient’s history and their cough characteristics may flag suspicion of the alternative diagnosis, primary vagal neural hypersensitivity. In this regard, individuals ultimately diagnosed with refractory chronic cough are often significantly older (late 50s and 60s) than those with chronic cough secondary to an identifiable primary condition, and they often have markedly longer cough duration with evidence of prior failed diagnostic tests and therapeutic interventions [35]. Features of cough hypersensitivity are common, including evidence of allotussia, paresthesia, somatic cough triggers or a positive Arnold’s nerve reflex, provides indicative evidence of sensory neural dysfunction [45, 74, 86, 87]. Related to this, a patient’s self-assessment of their cough severity may be high if primary vagal neural hypersensitivity is an underpinning cause [88]. Hypersensitivity symptoms or signs have also been associated with cough persistence or treatment responses [87, 89–91]. For example, in real-world post-market studies of gefapixant responsivity in Japan, responders typically had more severe pre-treatment cough, dry cough and evidence of laryngeal paresthesia. Validated hypersensitivity tools, such as the Cough Hypersensitivity Questionnaire or the Newcastle Laryngeal Hypersensitivity Questionnaire prove to be useful for assessing neural hypersensitivity [92, 93], whereas cough-specific and general health-related quality of life scores may not always be different between refractory chronic cough and other causes of chronic cough [35]. Thus, a combination of empirical diagnostic work up and therapeutic intervention outcomes along with patient/ cough characteristics would support adopting a trial of neuro-targeted therapies earlier in appropriate patients.

### Emergent Neuromodulatory Therapies

The current therapeutic options for targeting dysfunctional neural process in chronic cough are largely based on therapies that have proven efficacious in managing neuropathic pain (discussed above). Nevertheless, these warrant consideration for patients with difficult to manage cough due to suspected primary vagal neural hypersensitivity, and clinical guidelines for their use already exist [3, 21]. An exciting development arising from continued advancements in understanding chronic cough mechanisms is the progression through active drug development programs of a range of novel drugs targeting dysfunctional peripheral and central elements of the nervous system [60, 61, 94]. Several molecules are in late-stage development and are poised to become new effective targeted therapeutic options for managing primary vagal neural hypersensitivity. Improved anesthetic drugs blocking vagal sensory neuron sodium channels are in development [95], along with drugs targeting P2 × 3 receptors expressed by vagal cough-evoking sensory nerve fibers [96, 97].

Advancements have also been made in centrally-acting neuromodulator antitussive drugs. The non-narcotic kappa-opioid receptor agonist/ mu-opioid antagonist, nalbuphine, demonstrated strong antitussive activity in recently completed phase II trials in both Idiopathic Pulmonary Fibrosis (IPF) cough and refractory chronic cough, and is now entering phase III programs [98]. Other drugs targeting central N-Methyl-D-Aspartate (NMDA) and Neurokinin 1 (NK1) receptors remain attractive given the role of these central signalling pathways in chronic pain. Indeed, the neurokinin 1 receptor antagonist Aprepitant, available as an anti-emetic medication used to prevent nausea and vomiting caused by cancer chemotherapy, was shown to significantly reduce cough frequency in a randomized controlled trial of patients with lung cancer [99] while another centrally acting NK1 receptor antagonist, Orvepitant, improved subjective cough severity measures in a trial of refractory chronic cough [100].

### The future evolution of the Neuroanatomic Diagnostic Protocol

**Fig. 2:**
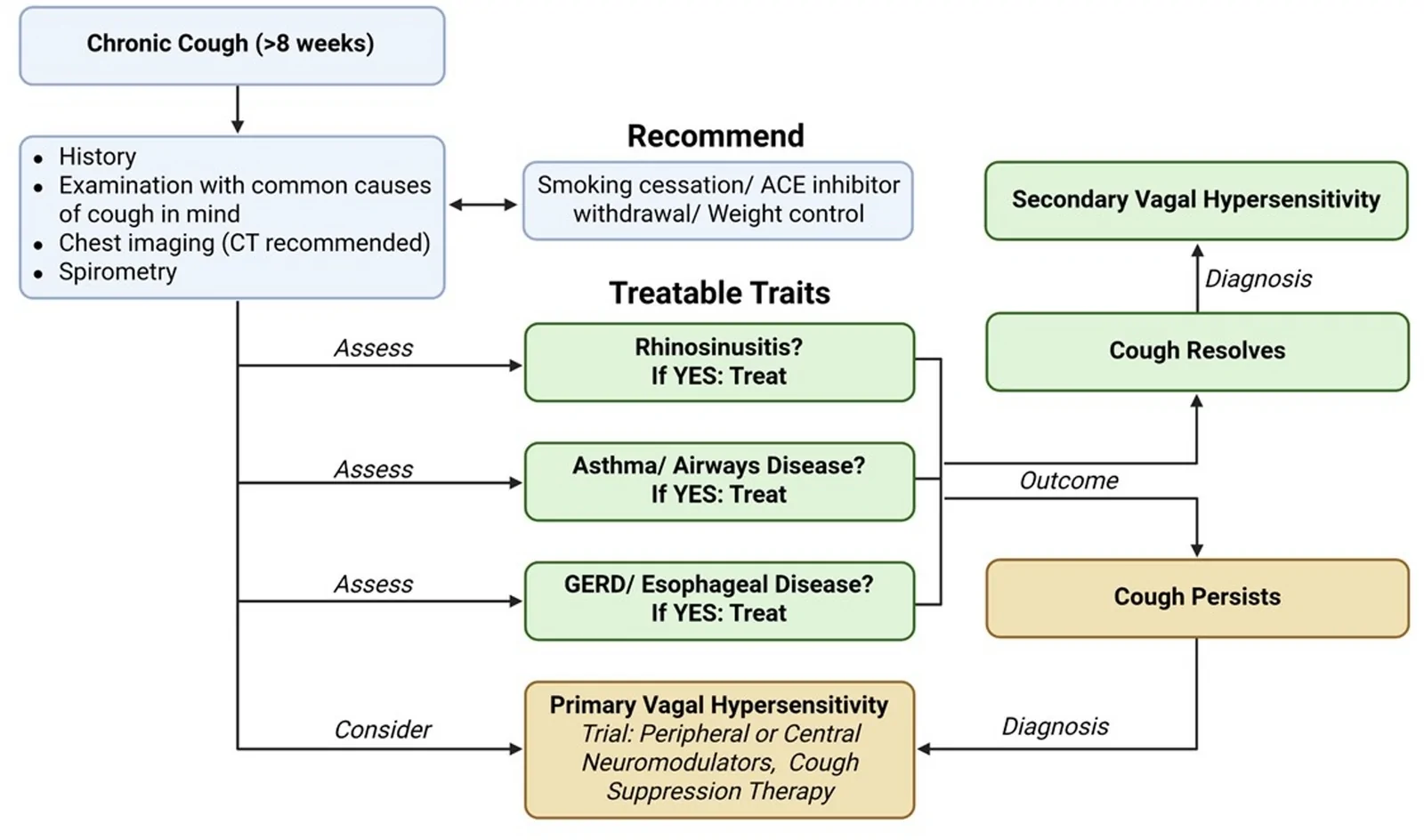
A conceptual roadmap for the application of the Neuro-Anatomic Diagnostic Protocol. The chronic cough patient requires detailed clinical work-up to carefully consider patient history along with potential red flags through appropriate chest imaging and lung function testing, perform etiology-based tests, and explore cough hypersensitivity phenotypes, as informed by clinical presentation. Smoking, ACE inhibitor therapy and weight control are important potential causal factors for modification. A clinician may reach diagnosis of primary vagal neural hypersensitivity either early in the diagnostic pathway based on these investigations, or alternatively, following trials of standard etiology-based therapies if secondary vagal neural hypersensitivity is first suspected but cough control is inadequate. Patients may conceivably have a mixed phenotype, requiring combinatorial intervention for primary and secondary vagal hypersensitivity

Then a question is raised: how should this new understanding be translated into an operational and highly effective clinical management algorithm? Within the original Anatomic Diagnostic Protocol, refractory chronic cough represents an operational end point, which is reached only after extensive investigations and sequential therapeutic trials of presumed peripheral causes. Anti-tussive therapies are typically reserved until late in the diagnostic pathway. Given the unmet needs of current clinical approaches, a Neuroanatomic Diagnostic Protocol should, if validated, enable selected patients to bypass prolonged sequential empirical therapeutic trials and receive earlier mechanism-based treatment. One potential strategy is to move beyond the operational label of refractory chronic cough and to assess treatable traits in parallel, allowing simultaneous evaluation and treatment of peripheral etiologies as well as hypersensitivity when clinically indicated. Another strategy is to identify clinical profiles or biomarkers that help predict patients with a predominant neural mechanism of cough and guide targeted treatments. With appropriate patient work-up, it is not unreasonable to confidently and safely diagnose primary vagal neural hypersensitivity early in the diagnostic pathway, significantly shortening the time to effective treatment (Fig. 2). It may also be reasonable to identify features of both primary and secondary vagal hypersensitivity within a single patient, necessitating combinatorial interventional approaches.

Finally, the success of a new diagnostic protocol will depend not only on conceptual advances but also on robust clinical evidence. Future studies should identify patients most likely to benefit from novel therapies and determine whether earlier integration of neural mechanism-based therapies improves patient outcomes compared with the traditional sequential empirical approach.

## Data Availability

No datasets were generated or analysed during the current study.
